# Lithotomy, (a Novel Case)

**Published:** 1868-04

**Authors:** Edward Whinery

**Affiliations:** Ft. Madison, Iowa


### LITHOTOMY--A NOVEL CASE.

*A Lead Pencil taken from the Urinary Bladder.*

Read before the Iowa State Medical Society, at its session in Davenport, May, 1867.

BY EDWARD WHINERY, M. D., OF FT. MADISON, IOWA.

On the 29th of November, 1866, Dr. M. Wahrer, of West Point, Lee County, Iowa, called on me, saying he had a case of stone in the bladder, in which he wished my assistance. On the following day I saw the patient, F. F., with him, at 9 o'clock a. m., and found the doctor was ready for the operation. He had sounded carefully the day before and satisfied himself that there was a calculus of large size. As his health was good, and the bowels free, he had administered an anodyne and required him to retain his urine then some 18 hours. We again introduced the sound, and I perfectly agreed with him that the stone was very large, and its presence very perceptible to the touch.

The mode of operation proposed and agreed upon was the lateral, and preparations being soon made, the operation was immediately commenced—I acting as assistant and hold- the staff. The doctor made his incision carefully, but boldly in view of the expected large calculus, but when the knife entered the bladder, there was not a free gush of urine as expected, until an effort was made to search for the foreign body. Upon introducing the finger, the doctor remarked that there was a foreign body, but it did not appear to be a stone. He made some effort to sieze it, but failing, desired me to examine it. The withdrawal of his finger probably caused it to be thrust forward so that the index finger of my left hand met it, and passing the forceps along its upper side, seized the foreign body, which proved to be a common lead pencil, nearly four inches long, and bluntly sharpened at one end. Nearly its whole surface was encrusted with a calculus deposit from a thin scale to about two lines in thickness, and being quite rough, gave us the idea of a large stone as the sound passed over its whole length. The patient was left in my charge to carry out the after treatment, and made a good recovery. He was operated on without chloroform or other anæsthetic, but was under the influence of an opiate, and we continued to exhibit sedative doses of morphine in combination with nitrate of potash, to have a constant antiphlogistic and sedative influence, so that he complained of no pain at any time during convelescence.

After ten days the urine passed off by the urethra, and in eleven days more he was well enough to take the cars for his home in Springfield, Ills.

After the operation he explained how the pencil got into the bladder.

He had been drinking and went to a house of ill fame, and supposes that he became thoroughly intoxicated—"dead drunk"—and that the prostitutes introduced it and passed it into his bladder. Of this, however, he knows nothing, but his trouble began immediately afterward.

